# Development of Bio-Nanofluids Based on the Effect of Nanoparticles’ Chemical Nature and Novel *Solanum torvum* Extract for Chemical Enhanced Oil Recovery (CEOR) Processes

**DOI:** 10.3390/nano12183214

**Published:** 2022-09-16

**Authors:** Karol Zapata, Yuber Rodríguez, Sergio H. Lopera, Farid B. Cortes, Camilo A. Franco

**Affiliations:** 1 Fenómenos de Superficie—Michael Polanyi, Facultad de Minas, Universidad Nacional de Colombia—Sede Medellín, Medellín 050034, Colombia; 2 Yacimientos de Hidrocarburos, Facultad de Minas, Universidad Nacional de Colombia—Sede Medellín, Medellín 050034, Colombia

**Keywords:** natural surfactant, bio-nanofluids, chemical enhanced oil recovery (CEOR), nanoparticles

## Abstract

This study aimed to develop novel bio-nanofluids using *Solanum torvum* extracts in synergy with nanoparticles of different chemical nature as a proposal sustainable for enhanced oil recovery (EOR) applications. For this, saponin-rich extracts (SRE) were obtained from *Solanum torvum* fruit using ultrasound-assisted and Soxhlet extraction. The results revealed that Soxhlet is more efficient for obtaining SRE from *Solanum torvum* and that degreasing does not generate additional yields. SRE was characterized by Fourier transformed infrared spectrophotometry, thermogravimetric analysis, hydrophilic–lipophilic balance, and critical micelle concentration analyses. Bio-nanofluids based on SiO_2_ (strong acid), ZrO_2_ (acid), Al_2_O_3_ (neutral), and MgO (basic) nanoparticles and SRE were designed to evaluate the effect of the chemical nature of the nanoparticles on the SRE performance. The results show that 100 mg L^−1^ MgO nanoparticles improved the interfacial tension up to 57% and the capillary number increased by two orders of magnitude using this bio-nanofluid. SRE solutions enhanced with MgO recovered about 21% more than the system in the absence of nanoparticles. The addition of MgO nanoparticles did not cause a loss of injectivity. This is the first study on the surface-active properties of *Solanum torvum* enhanced with nanomaterials as an environmentally friendly EOR process.

## 1. Introduction

Nowadays, chemical enhanced oil recovery (CEOR) processes are of primary importance for the oil and gas industry to increase the available reserves in mature fields [1,2]. Among the CEOR techniques, the injection of surfactants is of particular interest due to the ease of implementation in the field [1]. Generally, surfactant selection is based on reducing the oil–water interfacial tension as it improves the microscopic displacement of the oil phase based on the reduction of oil saturation [3,4]. However, there are other mechanisms such as wettability alteration and the viscosity of the displacement phase should be contemplated to increase the oil recovery [5,6,7]. The surfactants employed in the oil and gas industry are currently obtained through chemical routes, which could be costly and have negative environmental impacts such as low biodegradability and high toxicity [8,9]. For this reason, this industry needs to move towards the search for biosurfactants produced by microorganisms or from raw vegetal sources as these can be biodegradable, without toxicity to the ecosystem, and in most cases less expensive to produce [10,11,12,13,14,15]. In the late 1960s, microbes first produced biosurfactants as extracellular compounds through the fermentation of hydrocarbons. Most studies report the genera bacillus and pseudomonas as the main producers of biosurfactants, among which are rhamnolipids, lipopeptides, and surfactins like the most common biocompounds. These compounds have shown the ability to reduce interfacial tension, alter the wettability of porous media to water-wet systems, and improve oil recovery [16,17,18,19]. However, despite being safe, innovative, and environmentally friendly methods, there is great skepticism in the oil and gas industry regarding use of biotechnological methods for recovery processes. For example, if the microorganisms to generate the surfactant are injected into the reservoir, the cells could grow out of control and cause damage to the formation [20,21,22].

On the other hand, if biosurfactants are produced in ex situ conditions in great bioreactors, production costs and biotechnological logistics are difficult compared to commercial chemical surfactants, since being living systems, it is imperative to control the variables for the process such as temperature, pressure, availability of nutrients, among others, which again leaves biotechnological methods relegated despite being friendly to the environment [20,21]. Due to this, surfactant extracts from plant biomass for CEOR applications have been presented as profitable, reliable, and environmentally friendly methods [15,23,24,25]. Holmberg [26] defines natural surfactants as substances derived from renewable sources such as polyols [27], glucose, simple sugars, amino acid residues [15,26,27,28], lipids, and mixtures of these biomolecules isolated from different plant parts, such as leaves, flowers, roots, and seeds [29,30,31,32] with double chemical polarity, the species *Ziziphus Spina*, *Christy,* and *Jatropha curcas Linn* being the most widely used plants for the extraction of natural surfactants [33,34,35].

At the same time, there are multiple studies on the surface-active capacity of saponin and terpenoid-type molecules present in many unused vegetable sources as the waste of coffee, castor, cashew, cardanol, and coconut oils and plants of the genus solanum. Explicitly, the species *Solanum torvum* has reported a high content of saponins [36,37,38,39], but the most interesting thing is that in South America it is a perennial plant with flowers throughout the year, undemanding and useless, in fact invasive, capable of growing in disturbed sites, pastures, plantations, and roadsides, that can grow from 0 m above sea level (masl) to 1400 masl [36]. Moreover, many growers tend to eliminate it because it can cause damage to domestic animals due to its thorns and toxicity [40]. Given the background, *Solanum torvum* becomes a good candidate as a raw material for obtaining metabolites with tensoactive activity. Likewise, many surfactants have been potentiated with nanoparticles such as silica [41], magnetite [42], titanium [43], zirconium [44], alumina [45], and nanocapsules [46] for EOR.

Nevertheless, there are no reports of extracts from *Solanum torvum* for EOR applications in the absence or presence of nanoparticles of different chemical natures in the performance in EOR processes. This study aimed to develop new bio-nanofluids using *Solanum Torvum* extracts in synergy with nanoparticles of different chemical nature and to evaluate their performance as a sustainable proposal for EOR applications. For this, saponin-rich extracts (SRE) with tense-active capacity were obtained from fruits using ultrasound-assisted extraction (UAE) and Soxhlet extraction (SE) and characterized by Fourier transformed infrared spectrophotometry (FTIR), thermogravimetric analysis (TGA), hydrophilic–lipophilic balance (HLB), foaming capacity, saponin type by Liebermann–Burchard reaction, and critical micellar concentration (CMC). In addition, four bio-nanofluids using SiO_2_ (strongly acid nanoparticles), ZrO_2_ (acid nanoparticles), Al_2_O_3_ (neutral nanoparticles), and MgO (basic nanoparticles) and the best SRE extract were elaborated to evaluate the effect of the chemical nature of the nanoparticles on the SRE performance evaluated through static tests (interfacial tension, contact angle, viscosity, and capillary number) and dynamic tests (recovered oil curves and pressure drop curves) so that the greatest number of variables are considered for the use of this type of technology.

## 2. Materials and Methods

### 2.1. Materials

Fumed silica (SiO_2_) nanoparticles were provided by Sigma Aldrich (St. Louis, MO, USA) and aluminum oxide (γ-Al_2_O_3_) nanoparticles were provided by Petroraza S.A.S. (Sabaneta, Colombia), while magnesium oxide (MgO) and zirconium oxide (ZrO_2_) nanoparticles were synthesized according to the Sol-gel method following the procedures described by Perez-Robles et al. [47] with modifications as described below.

To obtain zirconium oxide (ZrO_2_) nanoparticles, the modified Sol-gel method was carried out. For this, 2 g of zirconium oxychloride octahydrate (ZrOCl₂ 8H₂O) and 0.21 g of calcium acetate monohydrate (C₄H₆CaO₄·H_2_O) were mixed in deionized water. The mixture was left under stirring at 100 rpm until coloration was transparent (Solution A). In parallel, 1.18 g of glycine were mixed with 14 mL of ethylene glycol at 1000 rpm (Solution B). Drops of B were added to solution A up to pH 4 to avoid the formation of precipitates during the polymerization reaction. The resulting mixture was stirred at 500 rpm for 24 h. Subsequently, it was heated to 80 °C to evaporate solvents and obtain a light brown solution, then the colored solution was cooled to room temperature, and finally it was calcined at 600 °C for 30 min [48].

To obtain magnesium oxide (MgO) nanoparticles, the modified Sol-gel method was carried out. Magnesium nitrate hexahydrate (MgNO_3_·6H_2_O) and sodium hydroxide (NaOH) were used to produce the MgO nanoparticles. A 0.2 M solution of MgNO_3_ 6H2O in deionized water was added drop by drop to 0.5 M solution of NaOH under magnetic stirring at 500 rpm. Then, a white MgO precipitate was formed. Stirring was continued for 30 min to ensure the reaction time. The precipitate was filtered and then washed with methanol to remove impurities, dried in a vacuum oven at 100 °C, and finally calcinated at 450 °C [47].

The point zero of charge (PZC), size particle, and thermogravimetric properties of the nanoparticles were estimated using instrumentation and protocols previously reported [49,50] and described below. Anhydrous acetic acid, methanol, hexane, chloroform, vanillin, sulfuric acid and oleanolic acid, zirconium oxychloride octahydrate, calcium acetate monohydrate, ethylene glycol, glycine, magnesium nitrate hexahydrate, and sodium hydroxide with degrees of purity ≥ 98% were provided by Sigma Brand (Sigma Aldrich, St. Louis, MO, USA). A Colombian light crude oil with 20°API gravity at 25 °C and a brine based on potassium chloride KCl [51] at 500 mg L^−1^ were used for the tests. The raw material used to obtain the extract rich in saponins (surfactant) was *Solanum torvum* purchased from a local market (Medellin, Colombia).

### 2.2. Methods

#### 2.2.1. Obtention of Saponin-Rich Extracts (SRE)

*Solanum torvum* fruits with no apparent diseases or lacerations were selected for the extraction of saponins. The pericarp was removed, dried at 70 °C, and crushed to between 1 and 5 mm. The methods used to obtain the extract enriched in saponins were conventional Soxhlet (SE) and ultrasound-assisted extraction (UAE). Methanol was used as an extraction solvent for both methods. After, liquid–liquid extractions with hexane were carried out to remove impurities.

For the Soxhlet extraction (SE), the method described by Wójciak-Kosior et al. [52] was used. The procedure is based on a system balloon–thimble–heater–condenser assembly. The sample:solvent mass ratio was 1:7, the extraction temperature was 80 °C, and the process ended after 24 h. Subsequently, the extract was evaporated, and the saponin-rich extracts (SRE-SE) were obtained and kept in the absence of oxygen at 70 °C.

For the ultrasound-assisted extraction (UAE), the method described by Wu et al. [53] with some modifications was used. For this, Elmasonic E30H ultrasound bath equipment with a frequency of 50/60 Hz and a sample:solvent mass ratio of 1:7 was used. The saponin-rich extracts (SRE-UAE) were obtained and kept in the absence of oxygen at 70 °C. The extraction temperature was set at 25 °C and the process ended after 2 h. Subsequently, the extract was vacuum filtered and the liquid fraction was evaporated. To eliminate non-polar impurities obtained from the previously described extractions and which do not correspond to saponin-type molecules (of double polarity), a complementary degreasing stage was added to each extraction method; for this, liquid–liquid extractions with hexane were carried out. The SRE-SE and SRE-UAE samples were previously reconstituted in distilled water and placed in a separating funnel with an equivalent volume of hexane (ratio 1:1). After 12 h, the phases were separated, and the aqueous fraction (pure extract) was dried in an oven in the absence of oxygen at 70 °C for 24 h.

#### 2.2.2. Characterization of SRE

##### Foaming Capacity of SRE

To evaluate the foaming capacity of the extract, the methodology proposed by Phillips et al. [54] was used. Solutions of 1 mL of deionized water and 0.05 g of SRE were mixed vigorously for 2 min. The appearance of stable foam on the surface of the liquid for at least 15 min indicates the surfactant capacity. Likewise, the content of saponins is associated with the height of the foam column (height less than 5 mm = no saponins detected; height of 5–9 mm = low content; 10–14 mm height = moderate content; and height greater than 15 mm = high content).

##### Nature of Saponins

The method proposed by Guo et al. [11] based on the Liebermann–Burchard reaction [12] allowed the determination of the type of saponins present in the sample. For steroidal saponins, the reaction becomes yellow–green, while for triterpenic saponins, the reaction will develop a pink–red coloration. For this, a small amount of extract was mixed with acetic anhydrous:chloroform in a 1:1 ratio. Then, three drops of concentrated sulfuric acid (72% wt.) with vanillin (0.5% wt.) were added, they were left to react for 10 min at 60 °C, followed by a cold-water bath for 15 min, and later colorimetric observations were made using an ultraviolet–visible (UV-VIS) spectrophotometer Multiskan SkyHigh Microplate Spectrophotometer (Thermo Scientific, Waltham, MA, USA).

##### Physicochemical Properties of SRE

To characterize the SRE extract, Fourier transformed infrared spectrophotometry (FTIR), thermogravimetric analysis (TGA), hydrophilic–lipophilic balance (HLB), and the critical micelle concentration (CMC) analyses were carried out, as described below.

FTIR was performed using an IRAffinity-1S spectrophotometer (Shimadzu, Torrance, CA, USA) according to the method proposed by Samal et al. [55] that aims to highlight chemical groups of the molecules present in the SRE extract. For this, approximately 1 mL of the SRE extract was deposited in the corresponding equipment compartment and the transmittance of the sample was then measured as a function of wavelength from 4000 to 450 cm^−1^ with a resolution of 2 cm^−1^. The spectrum was interpreted using information previously reported by Samal et al. [55].

TGA was used to evaluate the thermal stability of the SRE extracts using a Q50 thermogravimetric analyzer (TA Instruments, Inc., New Castle, DE, USA). For this, 25 mg of SRE extract were deposited in the corresponding compartment and subjected to a heating ramp from 30 to 800 °C, at a rate of 10 °C min^−1^ under a N_2_ flow rate of 100 mL min^−1^. At the same time, weight loss was recorded. Finally, the instrument produced the characteristic curve using the Software Q Series-[Q50-1459-TGA Q 50@Mfg-tga] [56].

For the application in CEOR technologies, the concentration of the surfactant is mostly established above the critical micellar concentration (CMC) to guarantee the distribution of the surfactant in the water/oil interface in the form of micelles. The CMC is the concentration at which the profile of a physicochemical measure changes. For the CMC calculation, different SRE dilutions between 0 and 5000 mg L^−1^ in distilled water were prepared and the surface tension using a KRÜSS GmbH tensiometer employing the Du Nouy ring method was measured for each one, following the procedures described by Sharma and Shah [57]. CMC is the SRE concentration at which the surface tension profile stops changing for the samples evaluated. The surface tension for the dilutions was determined by evaluating the force required by the ring (instrument) to break the fluid/air interface.

Finally, the hydrophilic–lipophilic balance (HLB) was determined following the method proposed by Chun and Martin [58]. For this, the interfacial tension between toluene and water with 0.1% wt. of SRE was measured using a KRÜSS GmbH tensiometer employing the Du Nouy ring method, then Equation (1) was applied to calculate the *HLB* value

(1) $$HLB=\frac{-\left(\gamma -45.7\right)}{2.36}$$

where γ (mN m^−1^) is the interfacial tension between toluene and a 0.1% wt. aqueous extract of SRE. Finally, the equation was obtained from Chun and Martin [58].

## 3. Results and Discussion

### 3.1. SRE Extract and Nanoparticle Properties

**Figure 1:**
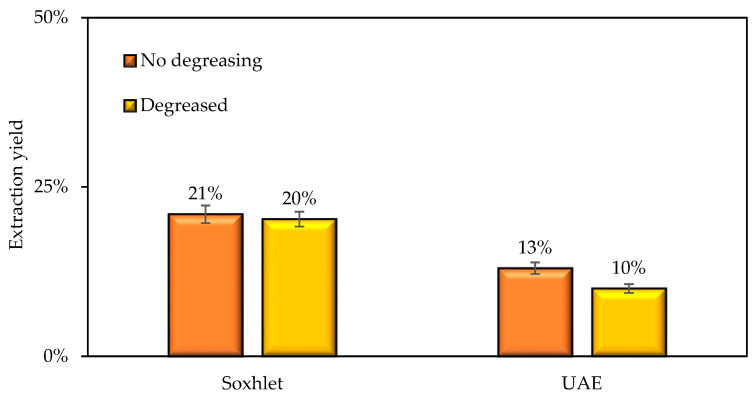
Extraction yield of Solanum torvum using Soxhlet extraction (SE) and ultrasound-assisted extraction (UAE) with and without a degreasing step.

The extraction yields of SRE from Solanum were 21% and 13% using the Soxhlet extraction (SE) and ultrasound-assisted extraction (UAE), respectively, and 20% and 10% coupled to both extraction methods, a subsequent degreasing process as reported in Figure 1. The results are attributed to the high temperature used in SE compared to UEA, that improves the diffusion of secondary metabolites from the vegetable matrix to the solvent during the extractive phases. Likewise, and unlike the UEA extraction, SE generates solvent recirculation on the sample, which allows, during the same process, to extract more and more secondary metabolites until solvent saturation, while the UEA extraction occurs in a single stage that can be insufficient considering the richness of non-polar compounds in the sample; other authors have found similar results [59,60]. Regarding the degreasing process, there are no significant changes in the extractive yields when the additional step is incorporated. These results demonstrate the absence of fatty acid in the *Solanum torvum* extract [61]. These results are essential since no additional purification steps are required to obtain extracts rich in saponins from *Solanum torvum*. The extracts obtained by SE were used for the static and dynamic tests.

Table 1 shows the results from the foam formation test and the Liebermann–Burchard reaction undertaken to confirm the presence and type of saponins obtained using the best method (SE). It is observed that the diluted extract of SRE after vigorous stirring forms a stable foam column (15 mm), indicating that is a saponin-rich sample according to the El Aziz et al. reports [62]. Secondly, the Liebermann–Burchard reaction evidenced the presence of steroidal-type Saponins due to yellow coloration. When the saponins are of the terpene type, they can react with vanillin (Liebermann–Burchard reagent) in an acidic medium and form red-colored adducts. On the contrary, if they are of the steroid type, the reaction does not take place, leaving the solution with the characteristic color of the Liebermann–Burchard reagent (yellow) [63].

**Figure 2:**
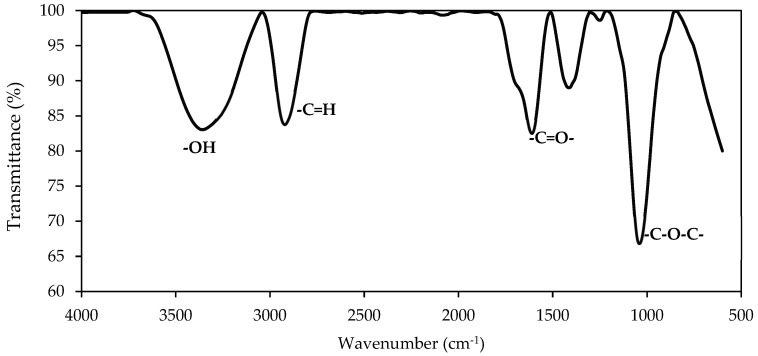
FTIR spectrum for SRE extract obtained from *Solanum torvum* using the SE method.

The FTIR spectrum of SRE-SE is shown in Figure 2. The band between 3500 and 3000 cm^−1^ is related to -OH bonds, the vibrations between 3000 and 2500 cm^−1^ are associated with the =C-H bond, the tension between 2000 and 1500 cm^−1^ is linked with the -C=O- and -C-C- bonds, while the vibrations around 1000 cm^−1^ are associated with -C-O-C- and -N-H- bonds. According to the studies carried out by Sarmah et al. [64], this spectrum is characteristic of the saponin structure composed of glycoside and steroid nuclei. Saponins are glycosides of steroids or triterpenoids, named for their soap-like properties: each molecule consists of a lipid-soluble element (the steroid or triterpenoid) and one that is soluble in water (sugar). The presence of these double polarity zones is associated with its surface-active properties and its foaming capacity [65].

**Figure 3:**
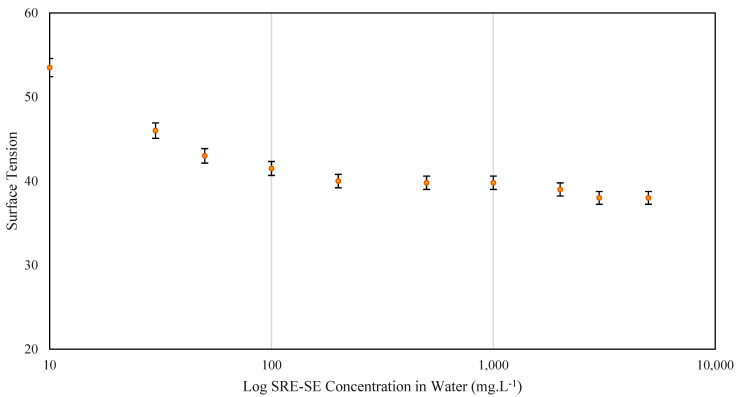
Critical micellar concentration (CMC) from surface tension determination for SRE extract obtained using the SE method at 25 °C.

Within the most important characterization tests for a surface-active extract is the CMC value and the HLB parameter that allows to determine the shape and chemical distribution of the sample in double polarity systems [66]. The results of these parameters are shown below. For the application in CEOR technologies, the concentration of surfactant is mostly established above the critical micellar concentration (CMC) to guarantee the distribution of the surfactant at the water/oil interface in the form of micelles [67]. The critical micelle concentration (CMC) for SRE-SE measured through surface tension is shown in Figure 3. According to the results, there is an inverse relationship between SRE-SE concentration in water and its surface tension. When the SRE-SE concentration is low, there are few saponin molecules that are separate from each other in monomeric form, favoring the interaction between water molecules by hydrogen bonds that increase the tension on the surface of the liquid. However, when the SRE-SE concentration increases it favors the formation of micelles that interact with the water molecules reducing the intense hydrogen bond forces and consequently the surface tension of the liquid [68]. By definition, CMC is the concentration after which there are no changes in the properties of a surface-active sample, and this concentration must be guaranteed operationally. The CMC value for the SRE-SE extract was 100 mg L^−1^, frequently commercial surfactants such as Tween 20 and Tween 80 have CMC values above 10,000 mg L^−1^; the difference between the CMC value of commercial surfactants and organic extracts is based on the complexity of the organic structures compared to the molecular structure of Tween-type surfactants. This molecular diversity allows greater interaction with water molecules, rapid changes, and at low concentrations the surfactant profile of the solvent. Finally, the use of concentrations between 100 and 2000 mg L^−1^ is suggested for surfactants applied in enhanced oil recovery (EOR) [57].

**Figure 4:**
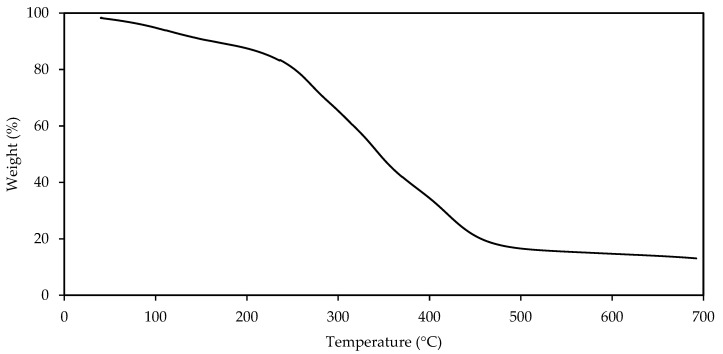
Thermogravimetric curve for SRE obtained using the SE extraction method (*n* = 3 ± 1 °C).

Alternatively, the hydrophilic–lipophilic balance (HLB) corresponds to an arbitrary scale of values used to classify surfactants between lipophilic -low HLB values [from 1–9] and hydrophilic -HLB values [10,11,12,13,14,15,16,17,18]. According to the results, the SRE-SE extract behaves as a hydrophilic surfactant with a solubilizing tendency (HLB = 15.97 ± 0.50). It has a high preference for the aqueous phase in a non-miscible mixture. The SRE-SE extract is analogous in hydrophilic terms to commercial surfactants such as Tween 20 and Tween 80, which report HLB values equal to 16.7 and 15, respectively. The results are interesting, considering that the injection phase of CEOR technologies is water-based, often with production water re-injected. The HLB value for SRE-SE assumes miscibility on the continuous phase and compatibility between the fluids to be incorporated into the reservoir [69]. Figure 4 reports the thermal stability of the SRE-SE extract. The results reveal that the mass loss at 150 °C was less than 15%. It is important to know the losses due to the thermal effect, to adjust the concentrations of the chemicals to be applied; however, minimum losses must be guaranteed to make the CEOR technologies techno-economically viable. In Colombia, for example, the typical temperatures of Colombian deposits do not exceed 120 °C. When comparing the decomposition temperatures, SRE-SE was analogous with surfactants used in the oil industry, presenting complete elimination at temperatures between 500 and 700 °C [70].

The physicochemical properties for the proposed particles are summarized in Table 2. The difference in the chemical nature of the nanoparticles was corroborated, especially strongly acidic, acidic, neutral, and basic structures were obtained, represented in fumed silica (SiO_2_), zirconium oxide (ZrO_2_), aluminum oxide (Al_2_O_3_), and magnesium oxide (MgO), respectively. The point of zero charge, PZC, is defined as the pH value at which the total net charge (external and internal) of the particles on the surface of the nanomaterial is neutral, that is, the number of positive and negative sites is equal [71]. On the contrary, taking the nanostructures to a pH far from their PZC guarantees that all the particles are homogeneously charged and increases the repulsion between them. This phenomenon brings advantages: avoiding interparticle attraction and the formation of large complexes that can block the throats’ reservoir pores [72]. Likewise, the correct dispersion of the nanoparticles in the injection phase improves the particle–saponin interaction, which is more likely to improve the surface-active properties of the extracts.

The results of the present investigation allow us to infer that under the typical pH of injection waters (6.7–7.5), no nanostructure will tend to self-aggregate. Likewise, the size of the individual structures in the nanometric regimen was corroborated by showing diameters less than 100 nm for at least 90% of the particles evaluated in each case (D90). Finally, the great thermal stability of all the synthesized nanoparticles was demonstrated, with mass losses of less than 5% at temperatures above 200 °C. The composition of the nanostructures is based on the ionic interaction between metals (Si, Zr, Mg, Al cations) and non-metals (O anions), forming ionic solids. According to the literature, ionic solids are of intermediate strength, extremely brittle, and have moderately high melting points, above 2000 °C [73]. In conclusion, this type of material is extremely stable at the temperature conditions of Colombian reservoirs, which, as mentioned above, do not exceed 200 °C.

#### 3.1.1. Effect of Nature and Concentration of the Nanoparticle

**Figure 5:**
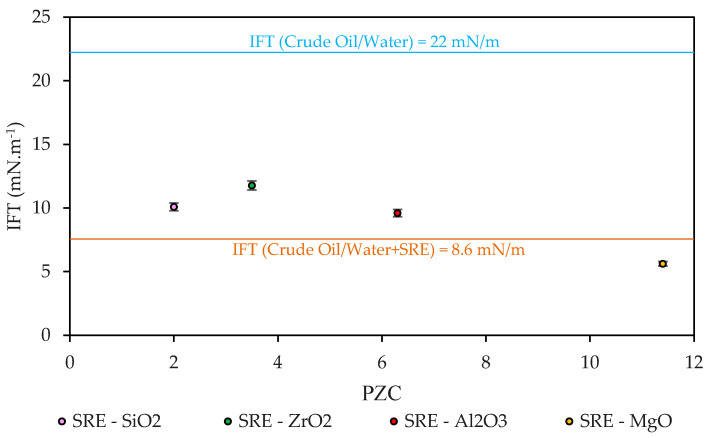
Effect of the addition of nanoparticles (300 mg L^−1^) on the interfacial tension (IFT) between crude and the aqueous extract SRE at 100 mg L^−1^.

Up to this point, all the nanoparticles seem to be advisable to incorporate into injection waters in terms of PZC and thermal stability. However, for the present investigation, the purpose was to identify the chemical nature of the nanoparticles that would improve the performance of the SRE-SE surface-active extracts. For this, four nanoparticles (Nps), such as SiO2 (strong acid), ZrO_2_ (acid), Al_2_O_3_ (neutral), and MgO (basic), were added to the SRE-SE extract to evaluate the effect of the interfacial tension (IFT). The interfacial tension (IFT) results between the Colombian crude oil and the four bio-nanofluids based on the different nanoparticles are presented in Figure 5.

**Figure 6:**
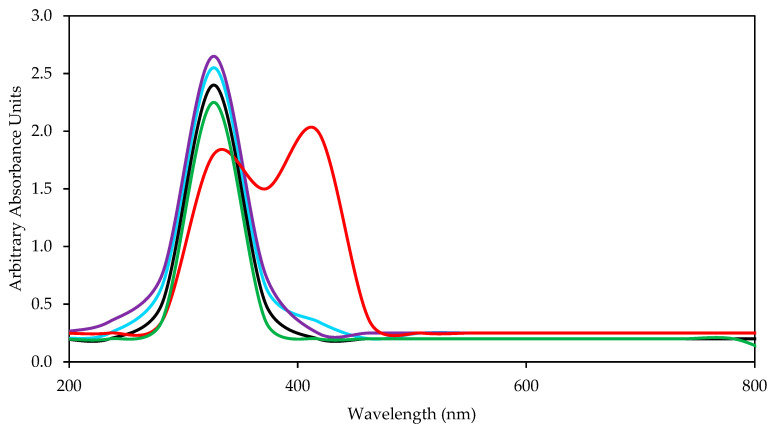
Effect of the addition of (Green Line) SiO_2_, (Black) ZrO_2_, (Blue Line) Al_2_O_3_, and (Red Line) MgO nanoparticles at 300 mg L^−1^ to the (Purple Line) aqueous extract SRE on the UV-Vis Spectrum and proposal of the MgO and Saponin interaction.

The interfacial tension between the water and the crude oil was 22 mN m^−1^, however, when 100 mg L^−1^ of SRE-SE were added to the water, the interfacial tension was reduced up to 61% thanks to the surface-active capacity of the extract obtained. Comparatively, the addition of MgO nanoparticles was the only favorable scenario, showing improvements in the surface-active capacity of the SRE extract of up to 34.6%. In contrast, the SiO_2_, ZrO_2_, and Al_2_O_3_ nanoparticles generated higher values of IFT when they were incorporated into the aqueous extract of SRE-SE (negative reductions). To highlight the main differences between the nanostructures, Figure 6 shows the relationship between the potential zero charges (PZC) of the nanomaterials and their ability to improve the surface-active capacities of SRE-SE, observing that acidic surface materials (PZC < 7.0) disfavor the surfactant capacity of the SRE-SE extract. To explain the results, consider the chemical composition of the nanomaterials. MgO nanoparticles were the only ones that presented a PZC point > 7. Thanks to this and considering the pH of the injection phase (SRE-SE aqueous = 7.3), MgO is the only material subjected to pH below its PZC, which causes a partial positive charge on its surface. This benefits the electrostatic interaction with nucleophile oxygen present in the water molecules by ion–dipole interactions, as other authors have pointed out (chemisorption of water on MgO) [74,75,76,77]. At that time, the lack of water molecules available for chemical interactions drives the saponin molecules to interact between themselves, forming micelles with a higher probability and thereby improving the tension between immiscible phases [78]. The interaction of the nanoparticles with water molecules was evaluated using a UV-Vis spectrum (Figure 6). The results showed that only the presence of MgO nanoparticles in the SRE-SE solution caused a shift in the spectrum for SRE-SE that indicated new molecular forms in the system [79]. This phenomenon is well described and is called chromic shift due to micelles formation [80,81,82].

In contrast, the increase in interfacial tension between the crude oil and the SRE-SE solution in the presence of the other nanoparticles is explained by considering the inability of these structures to interact with the water molecules due to their anionic forms at the working pH and also to the ability to adsorb saponin molecules, with which the monomers are engaged in the interactions with the nanostructures and do not form micelles decreasing the surface-active properties for SRE, what was verified through the spectra and the IFT measurements [83].

**Figure 7:**
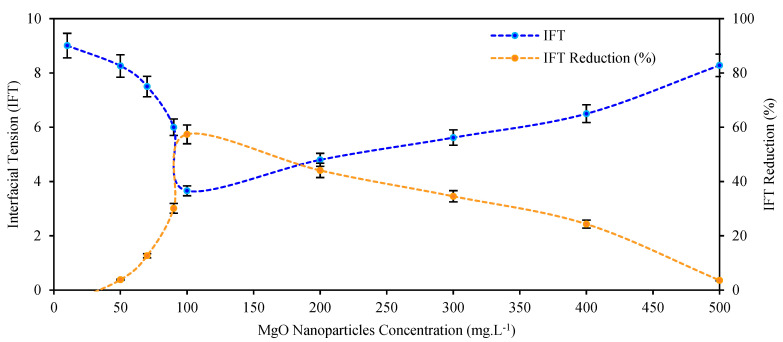
Effect of MgO nanoparticle concentration on the interfacial tension (IFT) between crude oil and the SRE solution.

For the best nanoparticle, MgO, 10 different concentrations were evaluated. The results revealed a relationship between the concentration in the SRE-SE solution and their capacity to improve the interfacial tension between the immiscible phases: 100 mg L^−1^ was the optimal concentration, causing interfacial tension reductions up to 57.4% compared to the SRE-SE solution without nanoparticles (Figure 7).

Some studies revealed that low concentrations of nanoparticles do not alter the surface-active properties of the extracts because of dilution effects; for the present case, concentrations lower than 100 mg L^−1^. However, after a certain concentration (greater than 100 mg L^−1^ for the present study), the interaction between the surface-active molecules (saponins) and the nanostructures by physisorption is favored, specifically interaction type π-electrons (saponins) and cations (Al, Zr, Mg and Si), which prevents the formation of micelles and thus reduces the efficiency of the surfactant [84,85]. Most chemical systems have a normal distribution in relation to the dose–response, that is, for most nanostructures there is an optimum concentration point at which the active properties of the system where they are incorporated improve; Many authors have reported this behavior [86,87]. For the present study that concentration is 100 mg L^−1^ of MgO nanoparticles.

In conclusion, the tenso-active properties are favored as long as the incorporation of new ingredients to the system favor the surfactant–surfactant encounter, so the nature and concentration of the nanostructures must be carefully considered.

#### 3.1.2. Capillary Number (Nc)

The CEOR technologies based on surfactants are not based on the improvement in the viscoelastic properties of the displacing phase, however, the additives of the present investigation favored the viscosity of the injection water up to 400%, which would improve the mobility ratio dragging a greater bank of crude using the piston effect (improvement in viscous forces). On the other hand, the IFT value for the evaluated systems revealed the capacity of the SRE-SE extract, accompanied by the MgO nanoparticles, to reduce the water/crude interfacial tension up to 79% for the preciously explained effects (improvement in capillary forces) (Table 3). The amount of hydrocarbon that is produced by natural energy of the reservoir or by the application of mechanisms such as the injection of additive water, is generally not greater than 40% of the original oil in place, due to the effect of viscous forces that prevent fluidity of the crude oil and capillary forces that retain the hydrocarbon in the porous medium [88]. For this reason, residual oil mobility is subject to a competition between viscous forces and capillary forces, which is expressed through the so-called capillary number, which is defined in terms of these properties as described above. Table 4 shows the value of the capillary number (Nc) for three displacing systems: water, SRE-SE solution (SRE-SE at 100 mg L^−1^ in water), and the optimal bio-nanofluid—hereafter simply named bio-nanofluid (SRE-SE solution and 100 mg L^−1^ of MgO nanoparticles).

The results revealed that the addition of SRE-SE improved the Nc of the water up to 9 times (1000% increments), while the addition SRE-SE accompanied by MgO nanoparticles enhanced the water performance up to 28 times (approximately 2700% increments). According to the literature, to achieve well-level effects and increments during the oil recovery process, the Nc improvement in the displacing fluid must be at least one order of magnitude (1000%). Nanoparticles can improve the performance of surfactants either by altering the wettability of the porous medium, to cause it to have less affinity for oil, or by improving the ability of surfactant molecules to form micelles and locate at the polar–nonpolar interface; in both cases, it is sought to favor the microscopic mobility of the crude oil bank. The capillary number allows both effects to be measured [41,89,90].

#### 3.1.3. Core Flooding Test

To simulate the behavior of the technology under reservoir conditions, displacement tests were carried out in porous media, under real pressure and temperature conditions. This will allow us to predict with a certain level of certainty the performance of the nanotechnology under reservoir conditions. The absolute permeabilities for Core 1 and Core 2, employed to evaluate the SRE-SE solution and the bio-nanofluid, respectively, were 13.5 and 12.1 mD. The effective permeabilities before and after the injection of each treatment are shown in Table 4. According to the results, crude oil mobility was improved by 8% and 17%, respectively, indicating that the bio-nanofluid has more activity towards the displacement of the oil phase.

**Figure 8:**
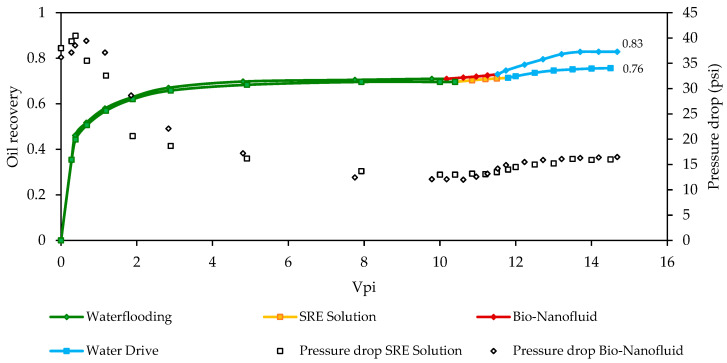
Recovered oil using SRE solutions and bio-nanofluids based on SRE and pressure drop for each scenario at 80 °C and 2500 psi (17.24 MPa) confining pressure.

Finally, the results of the recovery curves and the pressure profiles for the SRE-SE solution and bio-nanofluid are presented in Figure 8. The results show that after waterflooding, approximately 70% of oil is recovered for the two scenarios, corroborating that the rock samples have similar characteristics and guarantee the reliability of the proposed experimentation. However, after the SRE-SE solutions and the water drive injection, the recovery rises to 76%, while the bio-nanofluid recovery increases up to 83%. Regarding incremental recovery, the bio-nanofluid recovered twice as much oil after secondary recovery compared to the SRE-SE solution, approximately 41% of the oil available for tertiary recovery compared with SRE-SE that was able to recover only 20%.

The results also show that the bio-nanofluid has a lower tendency for adsorption as the slope in the recovery curve (~10 pore volume injected Vpi) is higher than for the SRE solution [91,92]. The dynamic results support the findings at the static level, where the capacity of the bio-nanofluids through the enhancement of the capillary number was evidenced. The comparison of the pressure profiles of the control scenario and the one enhanced with nanomaterials allowed to establish that the addition of MgO nanoparticles did not cause blockages in the porous medium. In Colombia, for every 100 barrels of oil equivalent that exist in the 257 producing fields, it is estimated that only 30% is recovered. The reasons are the quality of the hydrocarbons, types of reservoirs, and rocks it is made from. This condition is not unique in Colombia but includes all the oil fields in the world. Nevertheless, depending on the improved technology that is applied, it can reach up to 65% of recovery [88,93]. This research showed superlative recovery factors under relevant simulated conditions: pressure and temperature of the reservoir, petro-physical conditions of the porous medium, physicochemical conditions of the injection phase, which opens up a plausible possibility to increase national reserves through environmentally friendly models. Any additional points in the recovery factor would mean millions of barrels that would be added to the economic production indicators [94]. For this reason, this proposal aimed at the development for future implementation of new improved recovery techniques that allow the use of energy resources and the minimization of environmental impacts.

## 4. Limitations

The main limitation of this proposal for it to be scalable and applicable in the field is to guarantee the supply of *Solanum torvum* raw material based on the requirement in the field. Although the Solanum is a perennial plant with production during any time of the year at different heights, which is not used in human or animal nutrition that compromises food security, it is important to develop planting protocols, collections, and extractions that guarantee the continuous supply of the surface-active extract for injection in the field.

## 5. Conclusions

Today there are many reports on plant sources for obtaining surface-active extracts; likewise, there is sufficient evidence in relation to the use of nanostructures for the improvement of the tenso-active properties of plant extracts. However, there is no information related to the genus Solamun and the effect of nature and the concentration of nanostructures during the elaboration of bio-nanofluids that allow the development of technologies in a conscious way in relation to effectiveness, biodegradability, and production cost. This study aimed to develop new bio-nanofluids using *Solanum torvum* extracts in synergy with nanoparticles of different chemical nature and to evaluate their performance as a sustainable proposal for CEOR applications. For this, four bio-nanofluids using SiO_2_ (strongly acid nanoparticles), ZrO_2_ (acid nanoparticles), Al_2_O_3_ (neutral nanoparticles), and MgO (basic nanoparticles) and the *Solanum torvum* extract were elaborated to evaluate the effect of the chemical nature of the nanoparticles on the extract performance evaluated through static tests and dynamic tests. The results allowed us to conclude that *Solamun torvum* is a good source of natural surfactants and the extraction by Soxhlet and without degreasing are recommended to obtain pure surface-active extracts rich in steroidal saponins. Likewise, the nanoparticles improve the innate surface-active performance of *Solanum torvum*, as long as properties such as surface charge and concentration of the nanoparticles are considered. For the present case, the use of cationic nanomaterials such as MgO nanoparticles at 100 mg L^−1^ is recommended: under this condition, the nanoparticles compete and interact with water molecules, favoring the formation of micelles from saponin molecules, which is reflected in a lower interfacial tension of the *Solamun torvum*/crude system, better capillary number, and higher dynamic recovery factors. Specifically, the development of a bio-nanofluid allowed to improve the surface-active properties of *Solamun torvum*, observing increases of up to 2000% of Nc and tertiary recoveries up to two times more compared to the use of the *Solamun torvum* free extract under reservoir conditions. According to the results, the use of a natural surfactant with tailored nanomaterials can become a clean and cost-effective technology for chemical enhanced oil recovery (CEOR) as long as planting, harvesting, and extraction are guaranteed for the continuous supply of the extract. To the best of our knowledge, this is the first study on the surface-active properties of genus Solanum enhanced with tailored nanomaterials as an environmentally friendly CEOR proposal, that if the supply conditions of the extract were considered it could be used under field conditions. Likewise, this work constitutes a contribution to the scientific understanding since no information is found in relation to the effect of the chemical nature and the concentration of nanoparticles directed to the improvement of CEOR technologies based on the *Solamun* genus.

## Figures and Tables

**Table 1:** Test to confirm the presence and the type of saponins in the SRE obtained from *Solanum torvum* using the SE method.

	Result	Observation	
Foam formation	+	Stable for 1 h	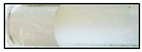
Liebermann–Burchard	+	Yellow coloration	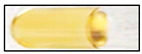

**Table 2:** Chemical and physical properties for nanoparticles used for modification of natural surfactants.

	Point Zero of Charge (PZC)	Size Particle (dp90) nm	Mass Loss at 200 °C (%)
Fumed silica (SiO_2_)	2.0 ± 0.1	68.0 ± 1.5	1
Zirconium oxide (ZrO_2_)	3.5 ± 0.1	66.0 ± 2.0	2
Aluminum oxide (Al_2_O_3_)	6.3 ± 0.1	61.4 ± 2.0	2
Magnesium oxide (MgO)	11.4 ± 0.1	69.4 ± 0.2	2

**Table 3:** Capillary number for the water, SRE solution, and optimal bio-nanofluid at 25 °C when used as the displacing fluid.

	Optimal Bio-Nanofluid	SRE Solution	Water
Contact Angle	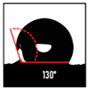	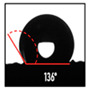	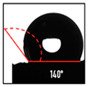
Viscosity	5.00 ± 0.01	4.37 ± 0.01	1.00 ± 0.01
IFT (mN m^−1^)	3.66 ± 0.12	8.60 ± 0.22	17.25 ± 0.20
Nc	1.80 × 10^−3^	4.00 × 10^−4^	1.00 × 10^−4^

**Table 4:** Effective permeabilities at residual saturations for surfactant solutions.

Property		Type of Treatment	
	Before	SRE-SE Solution	Bio-Nanofluid
Ko at Sw (mD)	14.3	15.5	16.8
Kw at So (mD)	3.2	2.7	2.9

## Data Availability

The data is included in the main text.
